# Relation of *AURKB* over-expression to low survival rate in BCRA and reversine-modulated aurora B kinase in breast cancer cell lines

**DOI:** 10.1186/s12935-019-0885-z

**Published:** 2019-06-18

**Authors:** Di Huang, Yu Huang, Zisheng Huang, Jiefeng Weng, Shuai Zhang, Weili Gu

**Affiliations:** Department of Surgery, Guangzhou First People’s Hospital, School of Medicine, South China University of Technology, No.1 Panfu Road, Yuexiu District, Guangzhou, 510180 Guangdong China

**Keywords:** Reversine, BRCA, Triple-negative breast cancer cells, AURKB, Aurora B, microRNAs

## Abstract

**Background:**

New therapeutic drug for breast cancer (BRCA), especially triple negative BRCA (TNBC), is urgently needed. Even though 2-(4-morpholinoanilino)-6-cyclohexylaminopurine (reversine) is an aurora kinase inhibitor, it also inhibits some cancer cells and human BRCA cells. However, the potential roles of reversine as a novel therapeutic agent for the treatment of BRCA remains unknown and must be further investigation. Thus, the relationship of reversine to aurora kinase in BCRA has not been reported. The relationship between AURKB and survival rate in BRCA has never been reported. Herein, we tested the roles of reversine on different BRCA cell line subtypes. We also investigated the relationship between AURKB and survival rate in BRCA as well as reversine to Aurora kinase expression in BCRA cell lines, including TNBC subtype, 4T1, MDA-MB-231, and luminal subtype MCF-7.

**Methods:**

Cell viability and apoptosis were detected using Cell Counting Kit-8 and flow cytometry analysis, respectively. Apoptotic and tumor-related proteins were tested using Western blot analysis. Important microRNAs that regulate BRCA were analyzed using RT-PCR. UALCAN public databases were used to analyze the targeted gene profiles, and the PROGgeneV2 database was used to study the prognostic implications of genes.

**Results:**

Reversine inhibits cell proliferation and induces cell apoptosis by modulating caspase-3 and bax/bcl-2 among the three cell lines. Data from the UALCAN public database show that BRCA tissues expressed high gene levels of *AURKB, TIMP1, MMP9*, and *TGFB1* compared with the normal tissue. Among the over-expressed genes in BRCA, *AURKB* ranks 9th in TNBC, 49th in luminal subtype, and 48th in HER2 subtype. High *AURKB* level in BRCA is highly related to the low survival rate in patients displayed in 18 databases searched via PROGgeneV2. The protein levels of aurora B kinase (Aurora B), which is encoded by *AURKB* gene, are highly suppressed by reversine in the three cell lines. The tumor-related proteins TGF-β1, TIMP1, and MMP9 are partially suppressed by reversine but with different sensitivity in the three cell lines. The reversine-affected microRNAs, such as miR129-5p, miR-199a-3p, and miR-3960, in MDA-MB-231 cell line might be the research targets in TNBC regulation.

**Conclusions:**

In BRCA, the level of *AURKB* are over-expressed and is related to low survival rate. Reversine contributes to anti-growth effect in BRCA cell lines, especially for TNBC, by modulating the aurora B. However, the invasiveness, metastasis, and anti-tumor effects of reversine in vivo and in vitro must be further investigated.

**Electronic supplementary material:**

The online version of this article (10.1186/s12935-019-0885-z) contains supplementary material, which is available to authorized users.

## Background

Breast cancer (BRCA) is the leading cause of cancer-related death among women [1, 2]. The incidence rate of BRCA among Chinese women increases rapidly with life style changes [3]. Even with the advances of current multidisciplinary therapies, including mastectomy, radiotherapy, and adjuvant chemotherapy, in treating BRCA, no effective systemic therapy has been established for metastatic BRCA. The overall 5-year survival rate for patients with aggressive BRCA is less than 25% after receiving systemic treatment [4, 5]. Therefore, efficient and promising therapies must be explored to reduce the risk of BRCA.

2-(4-Morpholinoanilino)-6-cyclohexylaminopurine (reversine) is a small synthetic purine analogue. Reversine primarily promotes myotube dedifferentiation derived from the murine myoblast cell line C2C12 [6, 7]. Reversine can facilitate the in vitro and in vivo differentiation of human fibroblasts into skeletal muscle cells [8]. Thus, reversine can reprogram somatic cells to a state of increased plasticity that can be manipulated to direct differentiation in different cell types. On the other hand, reversine displays an anti-cancer activity in various cancer cell lines, such as human acute or chronic myeloid leukemia cells [9, 10], multiple myeloma cells [11], human thyroid cancers [12], oral squamous carcinoma cells [13], human renal carcinoma cells [14], and urothelial carcinoma cells [15]. In BCRA, reversine induces cell cycle arrest, polyploidy, and apoptosis in human BRCA cells [16]. Reversine contributes to the growth inhibition of human BRCA cells through cell cycle arrest, polyploidy, and/or apoptosis induction [16]. However, the potential role of reversine as a novel therapeutic agent for the treatment of BRCA remains unclear. Recently, aurora kinases are new cancer therapy targets [17–19]. Furthermore, in Sessa’s study, reversine has been identified as a novel class of ATP-competitive aurora kinase inhibitor by forming the reversine-Aurora B kinase complex [9]. Therefore, we examined if reversine can inhibit BRCA cell lines by modulating aurora kinase B (Aurora B). Unexpectedly, the activity of reversine to aurora kinase in BCRA has not been reported. The relationship between AURKB and survival rate in BRCA has not been reported.

Therefore, in the present study, we first analyzed the role of reversine on three BRCA cell lines, including the TNBC subtype, 4T1, MDA-MB-231, and luminal subtype MCF-7. We then searched the gene *AURKB* expression in normal and BRCA tissues and its expressions in different BRCA cell types, including the Luminal, HER2, and TNBC subtypes. Furthermore, we analyzed the effect of AURKB gene level on the overall patient survival based on public databases. Finally, we analyzed the potential microRNAs affected by reversine for future interference experiment.

## Methods

### Cell lines and cell culture

BRCA cell lines, namely, MDA-MB-231, MCF-7, and 4T1, were purchased from Sun Yat-Sen University (Guangzhou, China). Cells were maintained in high-glucose Dulbecco Modified Eagle Medium (Gibco, USA) with 10% fetal bovine serum (Gibco, USA) supplemented with 100 U/mL penicillin and 100 µg/mL streptomycin in a humidified 5% CO_2_ incubator.

### Cell viability assay

Reversine (CAS NO. 656820-32-5) was purchased from Sigma-Aldrich (USA), which was dissolved in dimethyl sulfoxide (DMSO) (Sigma, USA) in accordance with the reagent instruction. Exactly 5000 cells were plated onto 96-well tissue-culture plates and grown in the above-mentioned medium. After overnight attachment, the cells were treated with medium only (containing 0.01% DMSO) as control or medium containing different concentrations of reversine. After incubation for 24 or 48 h, the number of metabolically active cells was determined using Cell Counting Kit-8 (CCK-8) assay (Dojindo, Shanghai, China). CCK-8 labeling reagent was added to the fresh medium, and the cell were incubated for 1 h at room temperature. Optical density value was examined at 520 nm by using a microplate reader (BioTek, USA). Results were analyzed through statistical methods in three independent studies. The percentage of cell viability was calculated relative to the control wells designated as 100% viable cells.

### Apoptosis assay and cell cycle analysis

Cells were seeded into six-well plates at a density of 3 × 10^5^ and cultured overnight. Cells were then treated with medium only (containing 0.01% DMSO) as control or different concentrations of reversine based on the data from the cell viability assay for 48 h. Annexin-V fluorescein isothiocyanate (FITC)/propidium iodide (PI) double staining kits (Keygen Biotech, Nanjing, China) was performed to detect apoptotic cells. Cells were washed with PBS twice and centrifuged at 1500 rpm for 10 min. Cell pellets were resuspended in 500 µL of staining buffer solution (5 µL of FITC and 5 µL of PI in 500 µL of binding buffer) and incubated for 15 min at room temperature in the dark. FITC or PI fluorescent intensities were analyzed using a flow cytometer (FACS Calibur, BD, USA), and 10,000 cells were evaluated in each sample. For the cell cycle analysis, after incubation with reversine for 48 h, cells were harvested and fixed in 70% ethanol overnight at 4 °C. After washing twice with PBS, cells were resuspended with 100 µL of RNase A, immersed in water bath at 37 °C for 30 min and then transferred into a tube containing 400 µL of PI-staining buffer (KeyGen Biotech, Nanjing, China). After incubation in the dark for 30 min at 4 °C, the treated cells were analyzed using FACS Calibur (BD, USA).

### Western blot analysis

Total cellular proteins were extracted from cells by using the Regulation of Investigatory Powers Act lysis buffer (Beyotime Biotechnology, China) and were quantified via bicinchoninic acid assay (BCA) by using the BCA protein assay kit (Beyotime Biotechnology, China). Approximately 40 µg of protein samples were subjected to standard SDS-PAGE and were transferred onto a polyvinylidene difluoride membrane (Millipore, USA). Nonspecific protein binding was blocked by incubating for 60 min in Tris-buffered saline, (TBS-T; 0.1% Tween-20) containing 5% (wt/vol) skim milk and 5% bovine serum albumin (BSA). Membranes were incubated at 4 °C overnight with primary antibodies (Additional file 1: Table S1) diluted in TBS-T with 5% BSA. After washing with TBS-T four times each for 10 min, the membranes were incubated for 60 min at room temperature with a second antibody (Proteintech, USA). After four additional washes with TBS-T, interest protein bands were detected by chemoluminescence method by using Clarity™ Western-enhanced Chemiluminescence Substrate Kit (BD, USA). The images were acquired from the chemiluminescence imaging equipment (GENE GNOME, USA), and scanning densitometry was performed using the Image pro plus software (IPP 6.0).

### Database searching for gene expression and survival analysis

UALCAN public database (http://ualcan.path.uab.edu/index.html) was used to analyze the previously reported targeted gene profiles [20]. The key words of the targeted genes, that is, *AURKB, TGFB1, TIMP1, MMP9, CASP3, BAX*, and *BCL2*, were entered in the gene symbol box, and the TCGA database for breast invasive cancer was chosen for analysis. The expression levels of the separated genes and the heatmap for query genes were acquired. The prognostic implications of genes were investigated using the PROGgeneV2 (http://genomics.jefferson.edu/proggene/) [21]. In this homepage, the single gene of AURKB was entered in the input gene box. Combined signature graphs only was selected, and BREAST was chosen for cancer type. Death was set as the survival measure, and the median was checked as bifurcate gene expression. A total of 19 databases were filtered automatically, and plots were created by selecting all. Exactly 18 databases were listed. The listed plots and indices of hazard ratio (HR), LCI (95%), UCI (95%), p value, and median survival were collected for analysis.

### RT-qPCR analysis

Total RNAs were extracted using TRIzol^®^ reagent (Invitrogen, Life Technologies, Carlsbad, CA, USA) in accordance with the manufacturer’s instructions. Extracted RNAs were transcribed into cDNA using the miScript II RT kit (Qiagen, Hilden, Germany). The reaction components were as follows: 1 µg of total RNA, 4 µL of RT mix, and 1 µL of 5 µM primer (C). RNase-free H_2_O was added to produce a total of 10 µL solution. The reaction was performed at 42 °C for 60 min and 72 °C for 10 min on an ABI PCR 9700 system (Applied Biosystems, Foster City, CA, USA). The RT-PCR system was set as follows: 3.6 μL of RNase-free H_2_O, 0.2 μL of 5 μM forward primer, 0.2 μL of 5 μM reverse primer, 5 μL of SYBR Green Surpermix, and 1 μL of cDNA sample. qRT-PCR was performed for the initial activation at 95 °C for 20 s, followed by 40 cycles at 95 °C for 10 s, 60 °C for 30 s, and 70 °C for 1 s. U6 was used as the internal reference gene, and the experiments were performed in triplicate. The 2^−ΔΔCq^ method was used for miRNA expression quantification.

### Statistical analysis

The data are presented as the mean ± standard deviation. GraphPad Prism 7.0 (Graph Pad Software, La Jolla, CA, USA) was used for statistical analysis. Statistical analysis was performed with one-way ANOVA. p < 0.05 was considered statistically significant.

## Results

### Reversine suppresses the cell proliferation and induces cell apoptosis and cell cycle arrest in vitro

**Fig. 1:**
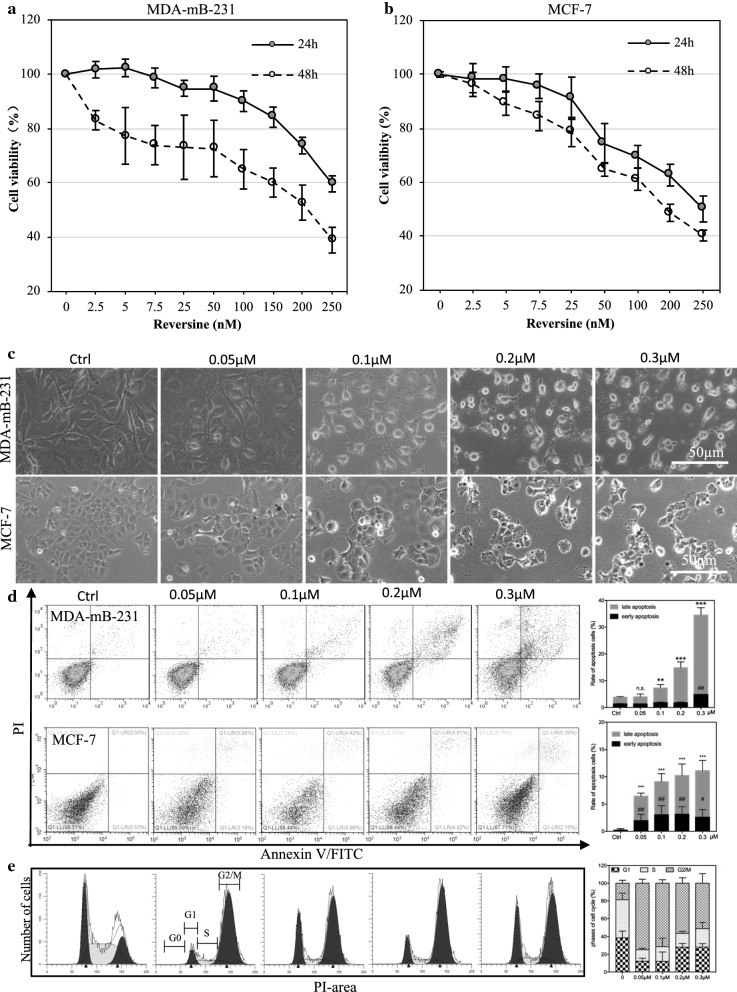
Reversine suppresses the cell proliferation and induces cell apoptosis and cell cycle arrest of human breast cancer cell lines. Time- and concentration-dependent changes of cell viability in the MDA-mB-231 (**a**) and MCF-7 cell lines (**b**) demonstrating the inhibition of reversion to human breast cancer cell lines. **c** Phase-contrast images of MDA-mB-231 (upper panel) and MCF-7 (low panel) in the indicated concentrations of reversine for 48 h. **d** Representative cell apoptosis phase images of MDA-mB-231 (upper panel) and MCF-7 (low panel) in the indicated concentrations of reversine for 48 h and the related apoptosis rate (%) in each cell lines (n = 3), compared with the control, **p < 0.01, ***p < 0.001, ^#^p < 0.05, ^##^p < 0.01 (one-way ANOVA), n.s., no significance. **e** Cell cycle analysis of MCF-7 in the indicated concentrations of reversine for 48 h and the related percent bar chart of each cell cycle stages (n = 3)

To confirm the effect of reversine on BRCA, we tested the cell proliferation rate in two subtypes of human BRCA cell lines. Reversine potently inhibited the cell growth of MDA-MB-231 and MCF-7 cell lines in time- and concentration-dependent manners (Fig. 1a, b). The half maximal inhibitory concentration (IC50) values at 24 and 48 h were 0.36 and 0.19 µM in MDA-MB-231 cells and 0.25 and 0.22 µM in MCF-7 cells, respectively. According to IC50 at 48 h, four concentrations of reversine, that is, 0.05, 0.1, 0.2, and 0.3 µM, were chosen for the following analysis in those two cell lines. Morphologically, reversine-treated cells changed from a normal appearance to rounded or swollen shapes with less cell-to-cell connections and high index of refraction. The cell number decreased with reversine treatment, showing the anti-proliferative effect of reversine on both MDA-MB-231 and MCF-7 cells (Fig. 1c).

Second, to assess the role of reversine on cell apoptosis, we performed annexin V-FITC/PI double-staining assay, and the apoptotic cells were computed by flow cytometry (Fig. 1d). The percentage of apoptotic cells, including early and late apoptotic cells, increased considerably in a concentration-dependent manner (Fig. 1d). By comparative analysis, we found that the potency of reversine to induce cell apoptosis was more evident in MDA-MB-231 cells than in MCF-7 cells at the same dosage. We then tested the cell cycle changes in MCF-7 cell (Fig. 1e). With increasing concentration, the cell rate in G1- and S-stages was drastically reduced and then slowly increased. Simultaneously, the cell rate in G2/M stage was drastically increased, indicating the function of reversine to induce cell cycle arrest in the G2/M stage.

**Fig. 2:**
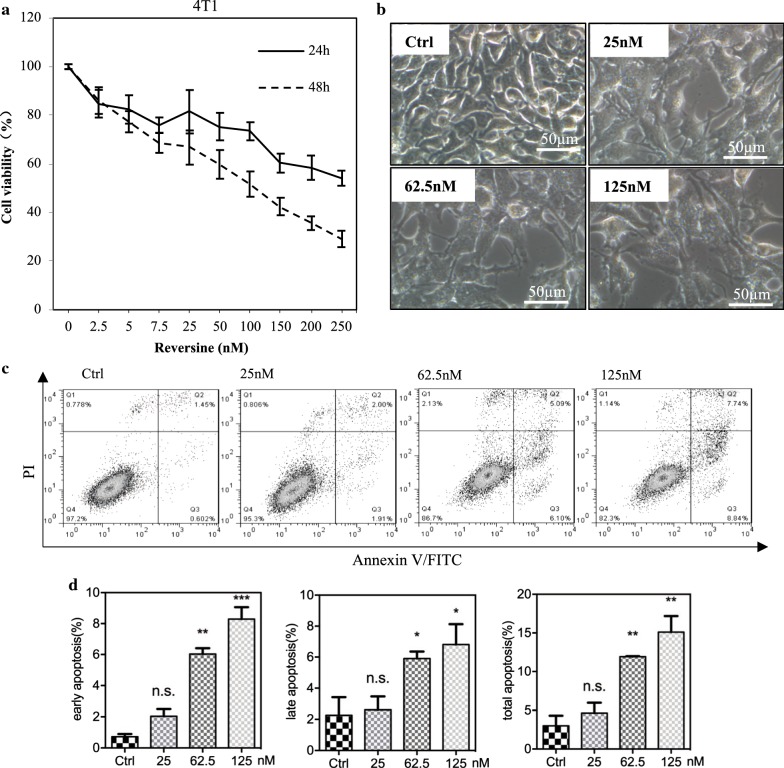
Reversine suppresses the cell proliferation and induces cell apoptosis of 4T1 mouse breast cancer cell lines. **a** Time- and concentration-dependent changes of cell viability indicating the inhibition of reversion to mouse breast cancer cell line. **b** Phase-contrast images of 4T1 cell line in the indicated concentrations of reversine for 48 h. **c** Representative cell apoptosis phase images of the 4T1 cell line in the indicated concentrations of reversine for 48 h. **d** Related apoptosis rate (%) in 4T1 (n = 3), compared with the control, *p < 0.05, **p < 0.01, ***p < 0.001 (one-way ANOVA), n.s., no significance

4T1 mammary carcinoma is a transplantable tumor cell line that is highly tumorigenic and invasive. The characteristics of 4T1 tumor has made it a suitable experimental animal model for human mammary cancer [22]. Thus, we tested the role of reversine on 4T1, a mouse TNBC cell line, in terms of cell proliferation rate and apoptosis (Fig. 2). Similar to the former results in human cell lines, reversine could suppress cell proliferation and induce cell apoptosis. The IC50 values at 24 and 48 h were 0.27 and 0.13 µM in 4T1 mouse BRCA cell line (Fig. 2a–d).

### Reversine induces apoptosis by modulating capsase-3 and bax/bcl-2 in BRCA cell lines

**Fig. 3:**
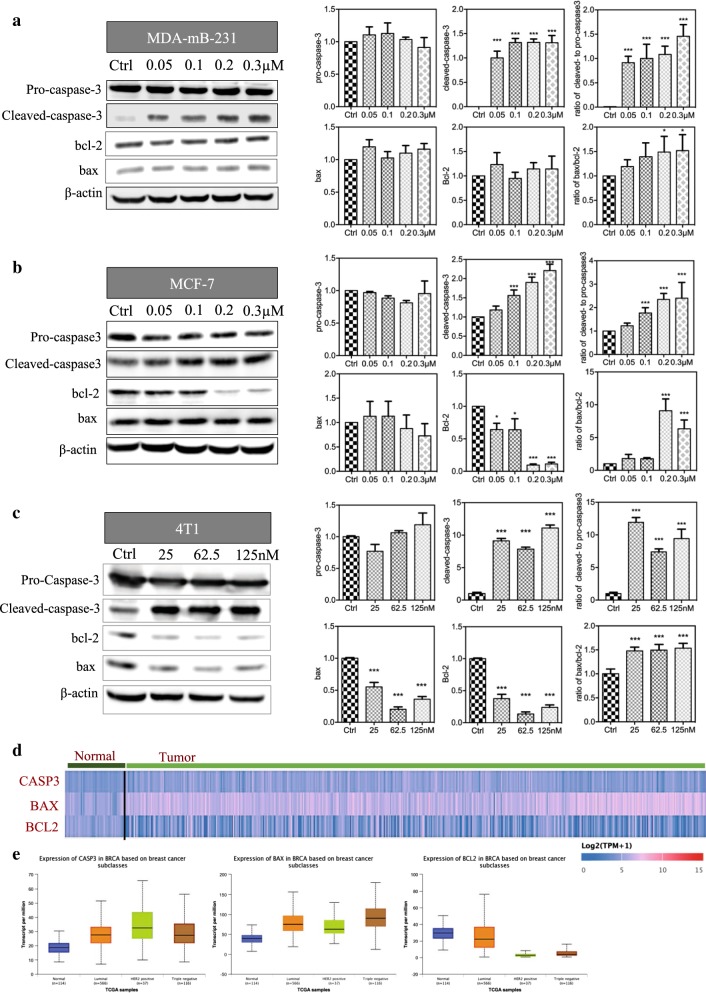
Reversine induces apoptosis by modulating capsase-3 and bax/bcl-2 in breast cancer cell lines. The levels of apoptosis-related proteins were tested with Western blot (left panel) analysis at the indicated concentrations of reversine for 48 h in MDA-mB-231 (**a**), MCF7 (**b**) and 4T1 (**c**) cell lines, and the relative expression of each proteins (left panels) was listed as bar grafts (n = 2) compared with the control, *p < 0.05, **p < 0.01, ***p < 0.001 (one-way ANOVA). **d** Heatmap of the three genes of CASP3, BAX, and BCL2 expressed in normal and breast cancer tumor tissues; data were acquired from UALCAN (see the materials and method for details). **e** Transcript expression levels of CASP, BAX, and BCL2 in BRCA based on breast cancer subclasses, indicating the over-expressed CASP3 and BAX genes and under-expressed BCL2 gene in the tumor tissues; data were acquired from UALCAN by analyzing the TCGA samples

To detect the protein level in reversine-induced apoptosis, we performed Western blot analysis (Fig. 3a–c). Data showed that the levels of cleaved caspase-3 increased in reversine-treated cells. The ratio of cleaved- to pro-caspase-3 was highly upregulated in the reversine treatment group in all of the three cell lines (p < 0.001). The expression of pro-apoptotic proteins (bcl-2 associated X) bax and anti-apoptotic protein bcl-2 were also determined. Quantitative analysis showed that the ratio of bax/bcl-2 was upregulated in high concentration groups in MDA-MB-231 and MCF-7 cell lines (Fig. 3a, b) and in all concentration groups in the 4T1 cell line (Fig. 3c). Hence, caspase-3 activation and bax/bcl-2 were related to reversine-induced apoptosis in BRCA cell lines. We then searched the different expressions of the apoptotic genes of CASP3, BAX, and BCL2 in BRCA via the UALCAN database (Fig. 3d). Compared with the normal tissues, gene CASP3 and BAX were highly expressed, and BCL2 had a low expression in BRCA tumors and in the luminal and TNBC subclasses (Fig. 3e).

### Reversine regulates tumor related proteins in BRCA cell lines

**Fig. 4:**
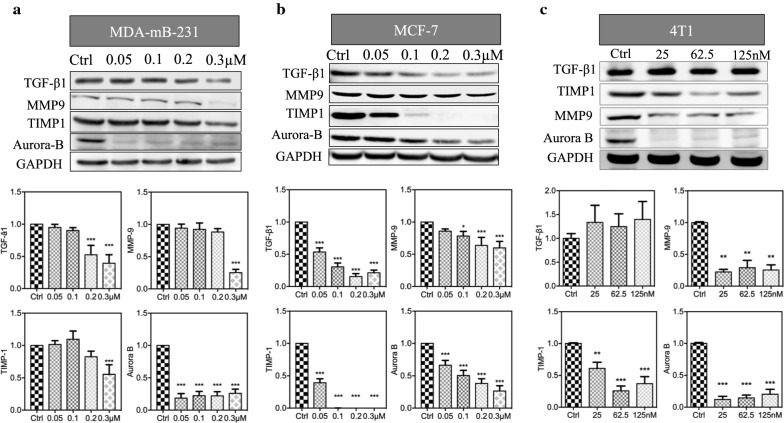
Reversine downregulates the expression of tumor-related proteins in breast cancer cell lines. The protein levels of TGF-b1, MMP9, TIMA1 m, and aurora-B were tested by Western blot analysis at the indicated concentrations of reversine for 48 h in MDA-mB-231 (**a**), MCF7 (**b**), and 4T1 (**c**) cell lines, and the relative expression of each proteins (inferior panels) was listed as bar grafts (n = 2) compared with the control, *p < 0.05, **p < 0.01, ***p < 0.001 (one-way ANOVA)

Aurora B is a cell-cycle-related protein, which is associated with tumorigenesis, making it a target for cancer therapy [17–19]. Reversine is an ATP-competitive aurora kinase inhibitor via forming reversine-aurora B kinase complex [9]. Here, aurora B protein was downregulated in all reversine concentrations in the MDA-MB-231, MCF-7, and 4T1 cell lines (Fig. 4a–c). Data demonstrated that reversine showed the same sensitivity in regulating aurora B expression in the three BRCA cell lines.

We then tested the protein levels of TGF-β1, MMP9, and TIMP1 proteins, which were important for tumor metabolism and metastasis [23–28]. In the MDA-MB-231 cell line, the three proteins were downregulated under relatively high reversine dosage. In the MCF-7 cell lines, however, almost all reversine dosages could downregulate the three proteins, which indicated that reversine showed relatively high sensitivity in MCF-7 than in MDA-MB-231 cell line for TGF- β1, MMP9, and TIMP1 protein expressions. For the mouse 4T1 cell line, reversine showed no effect on TGF-β1 protein but showed high suppression to TIMP1 and MMP9 in all indicated dosages.

### Over-expression of aurora B relates to lower survival rate in BRCA

**Fig. 5:**
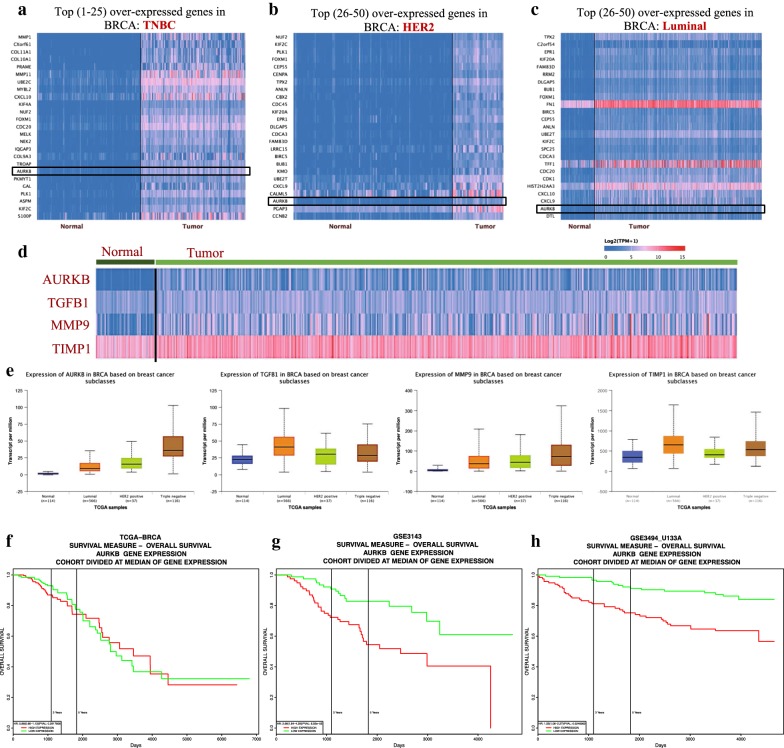
Overexpression of aurora B relates to lower survival rate in BRCA The heatmap of over-expressed genes in TNBC subtype (**a**), HER2 subtype (**b**), and luminal subtype (**c**), showing that AURKB genes ranked 19, 48, and 49 in the TNBC, HER2, and luminal subtypes. **d** Heatmap of the four tumor-related genes of AURKB, TGFB1, MMP9, and TIMP1 expressed in normal and breast cancer tumor tissues; data were acquired from UALCAN. **e** Transcript expression levels of AURKB, TGFB1, MMP9, and TIMP1 in BRCA based on breast cancer subclasses, indicating the over-expressed AURKB, TGFB1, MMP9, and TIMP1 genes in the tumor tissues; data were acquired from UALCAN by analyzing the TCGA samples. The overall survival at different AURKB expression levels in the TCGA (**f**), GSE3141 (**g**), and GSE3493_U133A database (**h**), demonstrating the influence of AURKB level on patient survival; data were acquired form the PROGgeneV2

As reversine displayed the same sensitivity in downregulating aurora B in MDA-MB-231, MCF-7, and 4T1 BRCA cell lines, we then explored the *AURKB* gene expression in BRCA tumors (Fig. 5). By searching the public database, we found that *AURKB* gene was over-expressed in BRCA tumors (Fig. 5d) as well as in the luminal, HER2, and TNBC tumor subtypes (Fig. 5e). Surprisingly, among the over-expressed genes, AURKB ranked 19, 48, and 49 in the TNBC (Fig. 5a), HER2 (Fig. 5b), and luminal BRCA subtypes (Fig. 5c), respectively. The median AURKB transcript per million were 1.247, 9.347, 15.605, and 36.086 in normal, luminal, HER2, and TNBC tissues, respectively, demonstrating the dramatic over-expression of AURKB, especially in the TNBC subtype. Accordingly, data acquired from PROGgeneV2 demonstrated the different survival probabilities at different AURKB expression levels in BRCA (Fig. 5e–h). To explain the detailed relationship between AURKB level and survival, we searched PROGgeneV2, which included 18 databases for BRCA (Table 1). Exactly 16 databases (88.89%) showed that patient with high expression of AURKB possessed higher hazard ration (HR > 1) than low expression (Table 1). The high expression population may die at approximately 2.14-fold of the rate per unit time compared with the low-expression population.

**Table 1:** Summary of the data form PROGgeneV2 by searching AURKB in the BCRA

Database	HR	LCI (95%)	UCI (95%)	p value	Median survival (d)	
					High level	Low level
TCGA	0.86	0.66	1.13	0.292	3462	2798
GSE7390	1.43	0.97	2.08	0.068	6255	–
GSR3494_U133B	1.54	0.34	6.93	0.573	–	–
GSR3494_U133A	1.55	1.06	2.27	0.025	–	–
GSE1456_U133B	3.18	0.85	11.88	0.086		–
GSE1456_U133A	1.85	1.21	2.83	0.004	–	–
GSE37751	1.15	0.65	2.06	0.632	–	2910
GSE42568	2.5	1.58	3.94	0.000	2456	–
GSE10893	2.2	0.55	8.83	0.266	1320	2220
GSE18229	2.28	0.59	8.8	0.231	1320	2220
GSE19536	1.13	0.75	1.69	0.555	–	–
GSE19783-GPL6480	1.5	1.01	2.21	0.042	3030	–
GSE21653	1.35	1.08	1.7	0.009	2920	3414
GSE2607-GPL887	5.78	10.8	30.97	0.040	900	–
GSE3143	2.66	1.64	4.29	0.000	2466	–
GSE48390	0.99	0.48	2.02	0.967	–	–
GSE58812	1	1	1.01	0.524	–	–
GSE6130-GPL887	5.61	0.95	33,19	0.057	1320	–
Mean	2.14	–	–	–	2544.9	2712.4

*BRCA* breast cancer, *HR* hazard ratio, *LCI* lower confidence interval, *UCI* upper confidence interval

### Reversine regulates expression prolife of microRNAs in different BRCA cell lines

**Fig. 6:**
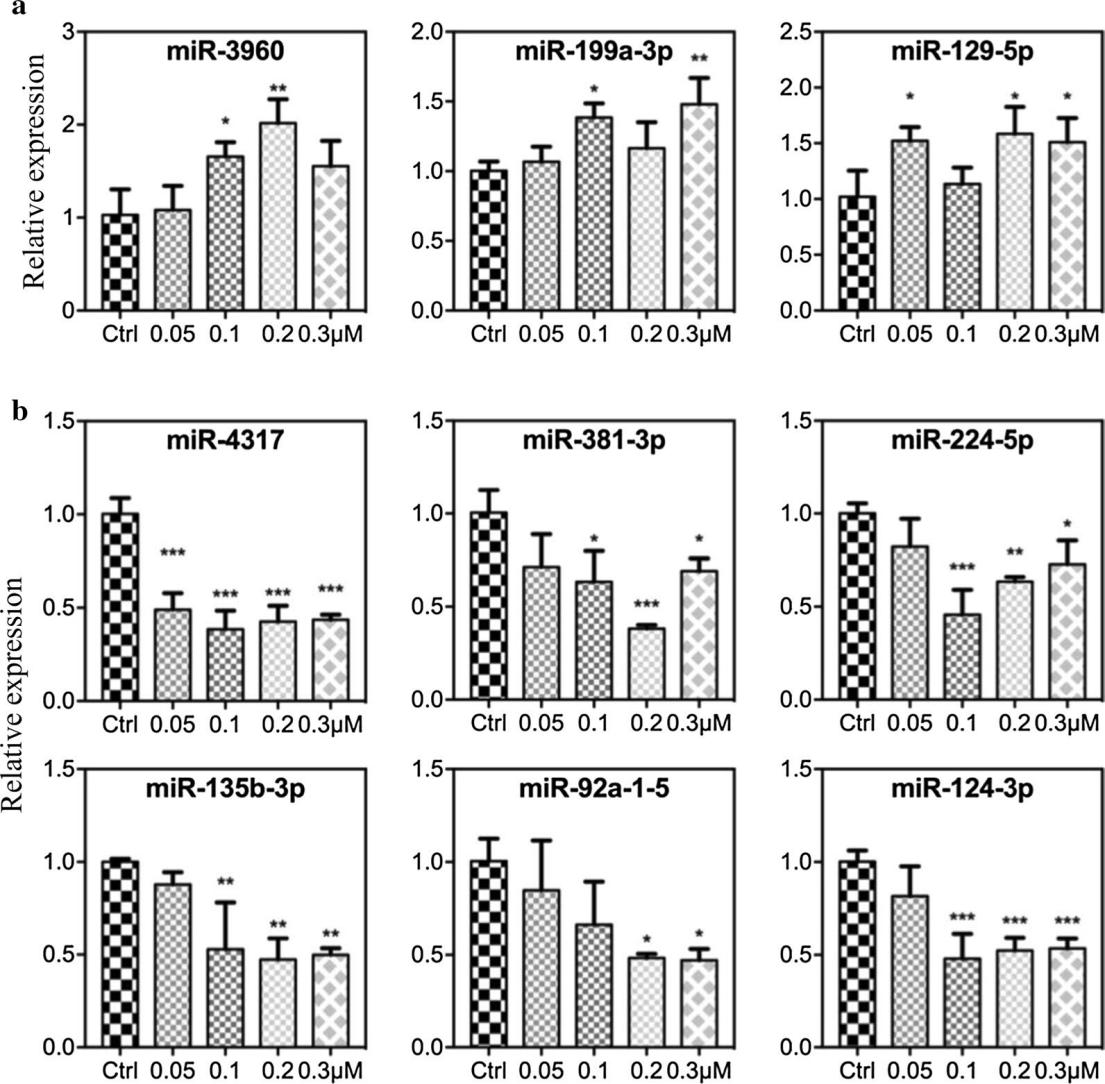
Reversine regulates the expression prolife of microRNAs in different breast cancer cell lines. **a** Three microRNAs were upregulated in the MDA-MB-231 cell line. **b** Six microRNAs were downregulated in the MCF-7 cell line, n = 3, values were determined relative to U6 and were presented as fold-change relative the levels in Ctrl, which was set as 1. *p < 0.05, **p < 0.01, ***p < 0.001 (one-way ANOVA)

Recent advances in the study of microRNAs indicate their important role in regulating cellular activities, such as proliferation, morphogenesis, apoptosis, and differentiation, by regulating the expression of various genes [29, 30]. For example, miR-129-5p suppresses BRCA proliferation by targeting CBX4 [31], and the downregulation of miR-129-5p via the Twist1-Snail feedback loop stimulates the epithelial–mesenchymal transition and is associated with poor BRCA prognosis [32]. Herein, we analyzed the role of reversine on microRNAs in BRCA. Reversine induced the upregulation of miR129-5p, miR-199a-3p, and miR-3960 in the MDA-MB-231 cell line (Fig. 6a) and the downregulation of miR-4317, miR-381-3p, miR-224-5p, miR-135b-3p, miR-92a-1-5, and miR-124-3p in the MCF-7 cell line (Fig. 6b). This data offered new content in our future research for further studying the regulating function of reversine on microRNAs in BRCA.

## Discussion

Reversine, a small molecule, which was first reported by Chen et al. [33] in 2004, can potentially induce the myogenic-lineage-committed cells to become multipotent mesenchymal progenitor cells [34]. Reversine can effectively reprogram somatic cells to a state of increased plasticity in different cell types [7, 35–41], promoting its great application value in regenerative medicine [42]. Reversine showed an in vitro anti-tumor effect to cancer cells [9–16], which demonstrated a potential anti-tumor application value. Thus, reversine is a potential chemotherapeutic agent that could be used against human BRCA cells.

First, by using CCK-8, a sensitive colorimetric assay for determining the number of viable cells in the proliferation and cytotoxicity assays, we found that reversine suppressed the growth of BRCA cell lines in a concentration-dependent manner. Possessing an anti-proliferative effect is the first step for screening and evaluating possible anti-tumor drugs. This CCK-8 data showed potential anti-tumor effect in BRCA cell lines. Subsequently, considering that cell apoptosis is one of the main mechanisms of anti-tumor drugs, we then tested the effect of reversine on cell apoptosis in the cell lines. Annexin V-FITC/PI double staining assay indicated that reversine-induced BRCA cell lines underwent apoptosis in a concentration-dependent manner. Western blot analysis results indicated that reversine induced cell apoptosis, which is related to the activation of caspase-3 and the regulation of bax/bcl-2 ratio in MDA-MB-231 cells. The in vitro data demonstrated the anti-proliferative and pro-apoptotic effect of reversine in BRCA cells [16].

Aurora kinases and serine/threonine kinases are essential for cell proliferation and play crucial roles in the regulation of multiple aspects of chromosome segregation and cytokinesis [43]. Reversine is a novel class of ATP-competitive aurora kinase inhibitor by forming the reversine-aurora B kinase complex [9]. Aurora kinases are targets of cancer therapy [17–19]. By combining these two concepts and our cell viability and cell apoptosis assay results, we speculated that reversine may inhibit the BRCA cell lines by modulating aurora B. We tested the protein level of aurora B in BRCA cell lines. As suspected, reversine downregulated the expression level of aurora B in the three indicated cell lines. Compared with the other three proteins of TGF-β1, MMP9, and TIMP1 that have functions in metabolism and metastasis, reversine showed high sensitivity for the inhibition of aurora B because all the selected concentrations of reversine could downregulate aurora B in the three cell lines. However, TGF-β1, MMP9, and TIMP1 were not regulated in all concentrations, and the concentration had no effect to the TGF-β1 level in 4T1 cell lines. To investigate the key role of AURKB, a gene encoding Aurora B, on BRCA, we searched the public databases to analyze the different levels of AURKB in normal and tumor tissues. Unexpectedly, the AURKB gene was over-expressed in BRCA tumors and in the subtype of luminal, HER2, and TNBC (Fig. 5e). Among the over-expressed genes, AURKB ranked 19th, 48th, and 49th in the TNBC, HER2, and luminal BRCA subtypes, respectively. The median AURKB transcripts demonstrated that AURKB was dramatically over-expressed, especially in the TNBC subtype. The over-expressed AURKB gene in BRCA are related to patient survival. Based on the survival data obtained from public databases, we found that the high-expression population may die at approximately twice the rate per unit time as the low-expression population. This finding showed us the potential target of *AURKB* in BRCA but must be further investigated via in vitro and in vivo AURKB silencing or knocking down. We are currently investigating this report.

## Conclusion

Collectively, our results suggested that the level of AURKB is overexpressed in BRCA and is related to low survival rate. Reversine contributes to anti-growth effect in BRCA cell lines, especially for TNBC, by modulating the aurora B. The in vivo and in vitro invasiveness, metastasis, and anti-tumor effects of reversine must be investigated.

## Additional file

**Additional file 1.** The information of the antibodies used in present work.

## Data Availability

The data supporting the conclusions of this paper are included within the manuscript.

## Abbreviations

BRCA: breast cancer
TNBC: triple negative breast cancer
HER2: human epidermal growth factor receptor 2
TGFβ-1: transforming growth factor beta 1
MMP9: matrix metallopeptidase 9
TIMP1: tissue inhibitor of metalloproteinases 1
Bcl-2: B-cell lymphoma 2
Bax: Bcl-2 associated X
CCK-8: cell counting kit-8
FITC: fluorescein isothiocyanate
PI: propidium iodide
RIPA: Regulation of Investigatory Powers Act
TBS: tris-buffered saline
HR: hazard ratio
ANOVA: analysis of variance
LCI: lower confidence interval
UCI: upper confidence interval
TCGA: The Cancer Genome Atlas
RT-PCR: real-time quantitative reverse transcription
SD: standard deviation
