# Supplementary material for: Relation of AURKB over-expression to low survival rate in BCRA and reversine-modulated aurora B kinase in breast cancer cell lines
Source: Cancer Cell Int. 2019 Jun 18;19:166. doi: 10.1186/s12935-019-0885-z (PMC6582545; doi:10.1186/s12935-019-0885-z)
Supplement: Supplementary file 1 — Additional file 1. The information of the antibodies used in present work. [file 12935_2019_885_MOESM1_ESM.docx]

**Additional Table S1**

The information of the antibodies used in present work.

| **Antibody** | **Source** | **Dilutions** | **Company, CAS NO.** |
| --- | --- | --- | --- |
| Aurora B | Rabbit | 1:1000(WB) | Novus Biologicals,NB110-55480 |
| Caspase-3 | Rabbit | 1:1000(WB) | Cell Signaling Technology, #9662 |
| Bcl-2 | Rabbit | 1:1000(WB) | Cell Signaling Technology, #2870 |
| Bax | Rabbit | 1:1000(WB) | Cell Signaling Technology, #2772 |
| MMP9 | Rabbit | 1:5000(WB) | Abcam,ab76003 |
| TIMP1 | Mouse | 1:1000(WB) | Abcam,ab1827 |
| TGF-beta1 | Rabbit | 1:1000(WB) | Abcam,ab66043 |
| GAPDH | Mouse | 1:3000(WB) | Proteintech Group,60004-1-Ig |
| β-actin | Mouse | 1:3000(WB) | Proteintech Group,60008-1-Ig |
| Peroxidase-conjugated Affinipure Goat Anti-Mouse IgG(H+L) | Goat | 1:3000(WB) | Proteintech Group, SA00001-1 |
| Peroxidase-conjugated Affinipure Goat Anti-Rabbit IgG(H+L) | Goat | 1:3000(WB) | Proteintech Group, SA00001-2 |

WB: Western blot.
